# Covid-19 Vaccination and the issue of insurance status: Daniel Schröpfer

**DOI:** 10.1093/eurpub/ckac130.029

**Published:** 2022-10-25

**Authors:** D Schröpfer, R Fortunato, N Devaud, R Koppensteiner

**Affiliations:** Sozial-Gesundheitliche Dienste, City Zuerich, Zurich, Switzerland

## Abstract

**Issue:**

It is estimated, that 11000 - 14000 people that live in Zurich do not have health insurance (∼3% of pop.). Covid-19 vaccination in Switzerland required an online registration (‘Vacme') with name, address and health insurance number. Health insurance in Switzerland is only accessible to people with registered residence in the country. This systematically excluded people without right of residence, health insurance or internet access.

**Description:**

To address this issue the health service of the city Zurich organized a vaccination site for the uninsured from 14.06.2021 onward. To achieve this a simplified registration procedure was established, which allowed us to offer Vaccination to everybody, independent of their insurance status. Instead of the health insurance number, on site personal used a dummy number and the address of our site to register those patients. To remove language and technological barriers. In order to lower, the inhibition threshold for people without legal residence the Police department was informed and agreed not to circulate the area of the vaccination site on vaccination days.

**Results:**

Between 14.06.2021 and 19.02.2022, 880 people came for vaccination of which 603 (69%) were clearly identifiable as individuals from non-insured populations. (See Graph)

**Lessons:**

It is therefore critical not to forget these people when organizing public health measures, especially when addressing a pandemic or other infectious diseases (HIV, Hepatitis). A group specific information approach is of paramount importance to reach such subpopulations. Non-insurance is a known barrier for universal access to care.

Acknowledgements: We would like to thank the involved departments of the city Zurich, chief medical officer canton zurich, our collaboration partners and the staff of our vaccination site.

**Key messages:**

- When fighting a pandemic it is of utmost importance to reach as many people as possible with given interventions including vaccination.
- Non-insurance is a known barrier for universal access to care.

